# Preventive Activity against Influenza (H1N1) Virus by Intranasally Delivered RNA-Hydrolyzing Antibody in Respiratory Epithelial Cells of Mice

**DOI:** 10.3390/v7092863

**Published:** 2015-09-21

**Authors:** Seungchan Cho, Ha-Na Youn, Phuong Mai Hoang, Sungrae Cho, Kee-Eun Kim, Eui-Joon Kil, Gunsup Lee, Mun-Ju Cho, Juhyun Hong, Sung-June Byun, Chang-Seon Song, Sukchan Lee

**Affiliations:** 1 Department of Genetic Engineering, Sungkyunkwan University, 2066, Seobu-ro, Jangan-gu, Suwon 16419, Korea; seungchan1007@gmail.com (S.C.); hmphuong2802@gmail.com (P.M.H.); sungle89@gmail.com (S.C.); happykke6@naver.com (K.-E.K.); meitantei007@naver.com (E.-J.K.); asteroid1975@gmail.com (G.L.); munju2004@naver.com (M.-J.C.); rsef741@gmail.com (J.H.); 2 Avian Disease Laboratory, College of Veterinary Medicine, Konkuk University, 120, Neungdong-ro, Gwangjin-gu, Seoul 05029, Korea; yellow0891@daum.net; 3 Animal Biotechnology Division, National Institute of Animal Science (NIAS), Rural Development Administration (RDA), 1500, Kongjwipatjwi-ro, Iseomyeon, Wanju 55365, Korea; pcs1778@korea.kr

**Keywords:** 3D8 scFv, antiviral effect, influenza virus, intranasal administration, nuclease activity, respiratory mucosal layer

## Abstract

The antiviral effect of a catalytic RNA-hydrolyzing antibody, 3D8 scFv, for intranasal administration against avian influenza virus (H1N1) was described. The recombinant 3D8 scFv protein prevented BALB/c mice against H1N1 influenza virus infection by degradation of the viral RNA genome through its intrinsic RNA-hydrolyzing activity. Intranasal administration of 3D8 scFv (50 μg/day) for five days prior to infection demonstrated an antiviral activity (70% survival) against H1N1 infection. The antiviral ability of 3D8 scFv to penetrate into epithelial cells from bronchial cavity via the respiratory mucosal layer was confirmed by immunohistochemistry, qRT-PCR, and histopathological examination. The antiviral activity of 3D8 scFv against H1N1 virus infection was not due to host immune cytokines or chemokines, but rather to direct antiviral RNA-hydrolyzing activity of 3D8 scFv against the viral RNA genome. Taken together, our results suggest that the RNase activity of 3D8 scFv, coupled with its ability to penetrate epithelial cells through the respiratory mucosal layer, directly prevents H1N1 virus infection in a mouse model system.

## 1. Introduction

Influenza virus, a RNA virus in the family Orthomyxoviridae is an acute respiratory infectious agent that causes significant morbidity and mortality in annual epidemics and global pandemic outbreaks [1]. In 2009, the pandemic H1N1 influenza A emerged from Mexico and the United States [2,3]. During the initial phases of the 2009 H1N1 pandemic, the use of neuraminidase inhibitors for the prevention of influenza virus infection was effective when vaccines were not available [4]. However, seasonal and 2009 pandemic H1N1 influenza viruses that are resistant to these drugs have emerged and subsequently spread worldwide [5,6]. In addition, increased influenza activity was reported in North America and Europe and in several countries in Asia in 2014. Over the years, many mutant influenza viruses such as A(H1N1)pdm09 and A(H3N2) have been identified, posing a great threat to worldwide public health [7,8]. Therefore, there is an urgent need for the development of novel antiviral therapeutics against new influenza viruses or their mutants.

3D8 scFv is an anti-DNA/RNA antibody that binds and hydrolyzes nucleic acids without significant sequence specificity [9]. This antibody was originated from an autoimmune-prone MRL-*lpr/lpr* mouse [10]. The 3D8 scFv protein was initially purified from *E. coli* and was subsequently shown to penetrate into the cytosol of HeLa cells via caveolae-mediated endocytosis [11]. Importantly, 3D8 scFv exhibits antiviral effects against herpes simplex virus (HSV), pseudorabies virus (PRV) and classical swine fever virus (CSFV) for prevention in transgenic HeLa and PK15 cells respectively [12,13]. In addition, 3D8 scFv also therapeutically protected RAW264.7 cells, macrophages of mouse, against murine norovirus (MNV) infection [14]. Based on these findings, it is clear that 3D8 scFv has antiviral effects against various DNA and RNA viruses in both *in vivo* and *in vitro* systems by penetrating into cells and directly catalyzing the hydrolysis of the viral genome.

Many infectious agents must enter the body at mucosal surfaces, and thus the mucosal layer functions as a first line of defense against infection [15]. Recently, the use of the nasal and pulmonary routes for the delivery of drugs and vaccines, especially against respiratory infections such as influenza, has attracted interest from pharmaceutical companies [16,17,18]. Numerous studies have investigated nasal delivery systems as a way to boost the host immune response as well as to deliver protein drugs [16,17]. Intranasal administration of a peptide of apoB-100 that was fused to the B subunit of cholera toxin (CTB) caused a 35% reduction in atherosclerosis in *Apoe*^−/−^ mice by the induction of regulatory T cells [19]. In another study, a nasal anthrax vaccine composed of nasal protective antigen (PA) fused with liposome-protamine-DNA (LPD) particles was developed for better protection against inhalational anthrax infection [20]. However, it is currently unknown whether protein drugs can efficiently pass through the respiratory mucosal layer to enter epithelial cells. Thus, verification of the ability of protein drugs to pass through the respiratory mucosal layer is important for the continued development of such approaches.

In this study, we tested the ability of intranasally administered 3D8 scFv protein to directly protect BALB/c mice against H1N1 influenza virus infection in the lung. Our results showed that administration of 3D8 scFv for 5 days prior to infection had strong antiviral effects against A/NWS/33 H1N1 influenza virus. To our knowledge, this is the first report showing that 3D8 scFv has antiviral effects against an RNA virus in an *in vivo* mouse model system through its intrinsic RNA-hydrolyzing activity coupled with its ability to penetrate into epithelial cells via the respiratory mucosal layer.

## 2. Materials and Methods

### 2.1. Animals

Six-week-old female specific pathogen-free (SPF) BALB/c mice (Orient Bio Laboratories, Seongnam, Korea) weighing 18–20 g were housed under standard laboratory conditions. All animal procedures performed in this study (permit number: KU15006) were reviewed, approved, and supervised by the Institutional Animal Care and Use Committee (IACUC) of Konkuk university.

### 2.2. Virus and Cell Culture

Madin-Darby Canine Kidney epithelical cells (MDCK cells) were provided by the Korean Cell Line Bank and were maintained in Eagle’s minimal essential medium (MEM) containing 5% fetal bovine serum (Hyclone, Logan, UT, USA), 100 U/mL penicillin- streptomycin (Hyclone) at 37 °C in a 5% CO_2_ atmosphere. Influenza A/NWS/33 (H1N1) virus (ATCC^®^ VR-219™) was purchased from the American Type Culture Collection (ATCC) and was grown in the allantoic sacs of 11-day-old chicken embryos at 37 °C for 2 days. The allantoic fluid was prepared as described previously [21]. For challenge studies, mice were anesthetized with an intraperitoneal injection of Avertin (375 mg/kg), followed by intranasal administration of 100 μL of 10^4^ EID_50_ influenza virus.

### 2.3. Virus Infection to MDCK Cells

MDCK cells were infected with 200 μL of 10^3^ EID_50_ influenza virus in serum-free DMEM for 40 min, washed, and incubated for 24 h in serum-free DMEM with trypsin (1 μg/mL). Cytopathic effects were observed by microscopy. Cells were lysed in TRIzol reagent (Molecular Research Center, Inc., Cincinnati, OH, USA) for RNA extraction. After generating complementary DNA, viral RNA expression in MDCK cells was determined using quantitative real-time PCR. All values were normalized against GAPDH cDNA using the 2^−∆∆*C*t^ method.

### 2.4. HA RNA Transcript Preparation and RNA Hydrolyzing Activity Test

Hemagglutinin (HA) cDNA was synthesized from total RNA isolated from H1N1-infected MDCK cells and cloned into pGEM^®^-T Easy, a vector that harbors the T7 promoter (Promega, Madison, WI, USA). The specific primers for HA were as follows: HA forward primer, 5′-ATG AAG GCA AAA CTA CTG GTC C-3′; HA reverse primer, 5′-AGT AGA AAC AAG GGT GTT TTT TCT-3′. HA RNA was synthesized from the cloned cDNA using an *in vitro* transcription kit (HiScribe T7 *In Vitro* Transcription; New England BioLabs, Ipswich, MA, USA) and incubated with 3D8 scFv purified protein (0.5 μg) for 1 h in TBS containing 2 mM MgCl_2_ at 37 °C. Reactions were terminated by addition of 10× loading buffer and analyzed by electrophoresis on 1% agarose gels and staining with ethidium bromide.

### 2.5. Purification of 3D8 scFv Protein

3D8 scFv protein was expressed in bacteria and purified by IgG-Sepharose affinity chromatography as described previously [9]. Protein concentrations were determined using an extinction coefficient for scFv of 1.995, in units of mg·mL^−1^·cm^−1^ at 280 nm, which was calculated from the amino acid sequence. Endotoxin content was determined using the Limulus Amebocyte Lysate (LAL) assay (PYROGENT^TM^ 25 single tests 0.125 EU/mL sensitivity, Lonza, Basel, Switzerland). The LAL assay was performed in pyrogen-free tubes to which 0.1 mL of 3D8 scFv protein (20 μg and 50 μg) and LAL reagent were added. After 1 h incubation at 37 °C, the tubes were observed by vertical inversion to see whether a stable solid clot was present or not. A visible solid clot was not observed in test tubes containing 3D8 scFv protein. The values of 3D8 scFv endotoxicity was <0.125 EU·mL^−1^.

### 2.6. Intranasal Administration of 3D8 scFv Protein

Mice were treated with 20 μg or 50 μg of 3D8 scFv protein for 3 or 5 days (Figure 2). PBS was given to the control groups for 5 days before challenge. After challenge with H1N1 influenza virus, clinical signs were observed daily for 14 days post-infection (p.i.). As a control, four mice from the group treated with 50 μg 3D8 scFv for 5-day were sacrificed on day 0 p.i., and lung samples were collected for histopathological examination.

### 2.7. Immunohistochemistry and Histopathological Examination

Lungs were collected from each group and fixed in 10% PFA. All samples were dipped in 4% sucrose in PBS, embedded, frozen, and sectioned using a cryomicrotome (Leica CM3050S, Wetzlar, Germany). The 10-μm-thick lung tissue sections for immunohistochemistry were mounted on silane-coated slides as described previously [22]. Tissue sections were incubated with rabbit anti-3D8 scFv and rabbit anti-HA primary antibodies (1:100 dilutions) for 1 h at room temperature. The tissue sections were incubated respectively with a TRITC-conjugated anti-rabbit secondary Ab (1:500 dilutions) to both anti-3D8 and anti-HA antibodies-incubated tissue section and visualized using a fluorescence microscope (Nikon Eclipse 80, Tokyo, Japan). For histopathological examination, samples were stained with hematoxylin and eosin (H&E) for microscopic examination and viewed under microscope (Nikon Eclipse E400).

### 2.8. Determination of Virus Titer

A lung from each mouse was homogenized in liquid nitrogen and resuspended in PBS. Measurement of the virus titer was performed as described previously [21].

### 2.9. Measurement of Cytokine and Chemokine Levels

Total RNA was extracted from lysed lung tissue using an Easy-spin Total RNA prep kit (Intron, Seongnam, Korea). cDNA was synthesized from 5 μg of total RNA using oligo-dT and Moloney murine leukemia virus (MMLV) reverse transcriptase (LexgeneBio, Cheongju, Korea). All primers were designed using the Primer 3 program [23] and are listed in Table 1. Levels of interferon-gamma (IFN-γ), tumor necrosis factor alpha (TNF-α), and interleukin-6 expression were determined by qRT-PCR as described previously.

**Table 1:** Specific primers for qRT-PCR.

	Forward	Reverse	Accession No
GAPDH	TGG CAA AGT GGA GAT TGT TGC C	AAG ATG GTG ATG GGC TTC CCG	NM_002046
3D8 scFv	TAT GCA CTG GGT GAA GCA GA	TGA GCT CCA TGT AGG CTG TG	AF232220 & AF232221
Hemagglutinin	CAC CCG TCT AGC AGT GAT GA	CTC AGT GCG AAA GCA TAC CA	U08903.1
Neuraminidase	CAC TTG GAA TGC AGG ACC TT	ACC AAG CAA CCG ATT CAA AC	HQ008256.1
TNF-alpha	CGT CAG CCG ATT TGC TAT CT	CGG ACT CCG CAA AGT CTA AG	NM_013693.2
IL-6	AGT TGC CTT CTT GGG ACT GA	TCC ACG ATT TCC CAG AGA AC	NM_031168.1
IFN-gamma	ACT GGC AAA AGG ATG GTG AC	GAC CTG TGG GTT GTT GAC CT	NM_008337.3

### 2.10. Statistical Analysis

All statistical analyses were performed using the GraphPad Prism software (GraphPAD Software). Kaplan-Meier survival curves were generated and compared using the Mantel-Cox log-rank test to determine statistical significance [24]. One-way ANOVA and Tukey’s *post hoc* *t*-tests were used for statistical analyses. Data are presented as means ± SEMs.

## 3. Results

### 3.1. Direct RNA Hydrolyzing Activity of 3D8 scFv against H1N1 Influenza Virus in MDCK Cells

**Figure 1:**
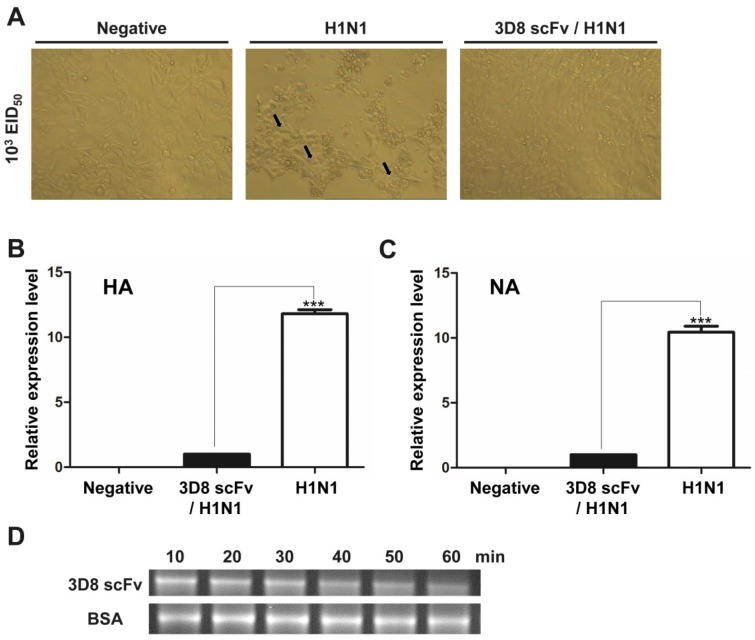
Direct catalytic activity of 3D8 scFv against H1N1 influenza virus. Madin-Darby Canine Kidney epithelical cells (MDCK cells) were infected with 200 μL of 10^3^ EID_50_ influenza virus for 4 h and then incubated for 24 h in serum-free medium with trypsin (1 μg/mL). (**A**) The cytopathic effects were examined by microscopy. Magnification 100×. The arrows indicated the cytopathic effects on host cells caused by H1N1 infection; (**B**) Transcripts of hemagglutinin and neuraminidase were measured by qRT-PCR and normalized by against GAPDH cDNA using the 2^−ΔΔ*C*t^ method. Data are shown as mean ± S.E.M of triplicate samples from three independent experiments. Data are mean ± standard error. *** Significantly different from 3D8 scFv/H1N1 group at *p* < 0.001 (one-way analysis of variance and Tukey’s *post hoc* *t*-test); (**C**) The RNA transcript of hemagglutinin was incubated with 3D8 scFv purified protein for 1 h; (**D**) Reactions were terminated at 10, 20, 30, 40, 50 and 60 min and analyzed by electrophoresis.

Based on research showing that 3D8 scFv could catalyze the viral genome and its transcripts [12], we tested the antiviral activity of endotoxin-free 3D8 scFv by treatment of purified 3D8 scFv proteins to MDCK cells. The cells were subsequently infected with 200 μL of 10^3^ EID_50_ H1N1 influenza virus in serum-free DMEM for 40 min, washed with PBS, and incubated for 24 h in serum-free DMEM with trypsin (1 μg/mL) in a 37 °C CO_2_ incubator. At 24 h post-infection, a less cytopathic effect (CPE) was observed under the microscope in the cells treated with 3D8 scFv compared with those treated with PBS (Figure 1A). The expression levels of hemagglutinin (HA) and neuraminidase (NA) were decreased approximately 10-folds in the 3D8 scFv-treated group compared with the PBS-treated group (Figure 1B,C). To determine the direct catalytic activity of 3D8 scFv against influenza virus, we tested the RNA hydrolyzing assay against the HA transcript of H1N1 influenza virus. Treatment with PBS for a prolonged incubation period did not result in degradation of mRNA, whereas purified 3D8 scFv protein resulted in an obvious time-dependent hydrolysis, as shown by a smeared mRNA pattern on a 1% agarose gel (Figure 1D).

### 3.2. Recovery from H1N1 Infection in Mice Treated with 3D8 scFv

**Figure 2:**
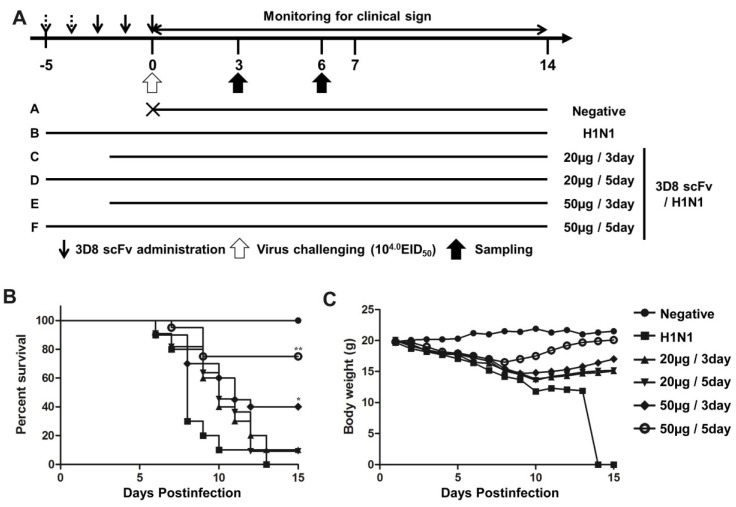
Antiviral effects of intranasally administrated 3D8 scFv on survival and body weight. (**A**) BALB/c mice were treated intranasally with 3D8 scFv protein (50 μg/mouse) for 3 or 5 days before infection with A/NWS/33; (**B**,**C**) Mice were monitored daily for 14 days to determine the rate of survival (**B**) and changes in body weight (**C**). Control group, *n* = 10; positive control group, *n* = 10; treatment groups, *n* = 10. Asterisks indicate significant differences (* *p* < 0.05, ** *p* < 0.01) compared with the positive (H1N1) control group (Fisher’s exact test).

**Figure 3:**
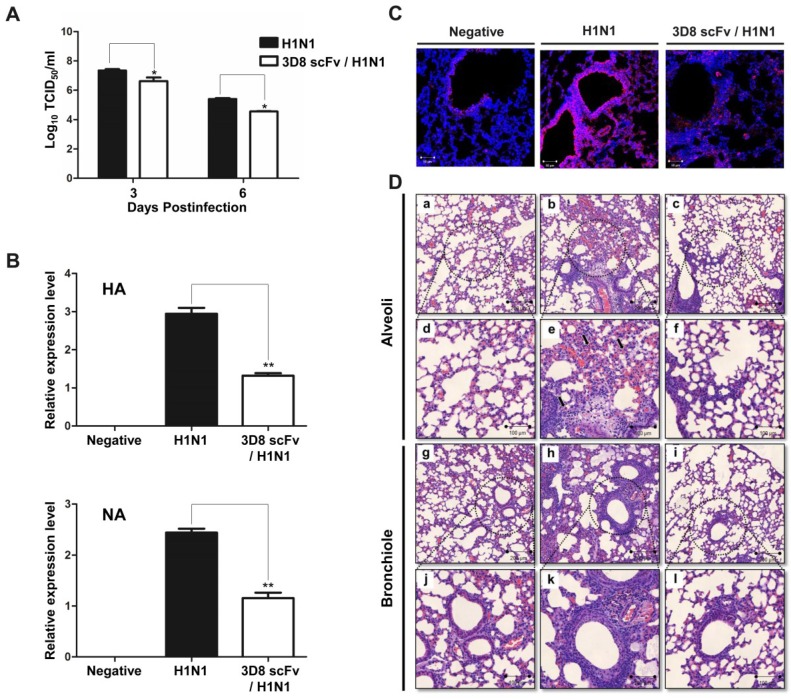
Preventative antiviral effects of 3D8 scFv protein in the lung. Emulsion samples were extracted from lung tissue after virus infection. (**A**) The viral titer was measured by TCID_50_ assay; (**B**) Viral replication was analyzed by qRT-PCR; (**C**) To confirm viral protein expression, H1N1 HA protein was detected by confocal microscopy using antibodies specific for HA and GAPDH. Asterisks indicate significant differences (* *p* < 0.05, ** *p* < 0.01) versus the positive (H1N1) control group (one-way analysis of variance and Tukey’s *post hoc t*-test); (**D**) Photomicrographs of lung sections in H1N1-infected mice treated with 3D8 scFv. Lung sections from mice at 3 days post challenge were stained with H&E. Uninfected lungs without treatment [panels (**a**,**d**), alveoli; (**g**,**j**), bronchiole]; infected lung without treatment [panels (**b**,**e**), alveoli; (**h**,**k**), bronchiole]; and infected lung treated with 3D8 scFv [panels (**c**,**f**), alveoli; (**i**,**l**), bronchiole].

To determine the protective effect of 3D8 scFv according to dose and number of injections, we pre-administered 3D8 scFv intranasally at two different doses (20 or 50 μg/day) for 3 or 5 days, and then challenged the mice with H1N1 influenza virus (Figure 2A). All of the mice in the control group were dead by day 13. In contrast, after 15 days we observed the survival rates of 50% and 70% in the groups treated with 50 μg/day 3D8 scFv for 3 or 5 days respectively. Likewise, mice that were pretreated with 20 μg/day 3D8 scFv for 3 or 5 days showed survival rates of 20% and 40%, respectively (Figure 2B). Weight loss in the control group progressed continuously after H1N1 influenza virus infection, whereas the weights of mice that were pretreated with 3D8 scFv decreased slightly after H1N1 influenza virus infection to normal by 8–10 days p.i. (Figure 2C). Overall, the group that was pretreated with 50 μg/day 3D8 scFv for 5 days exhibited the highest antiviral clinical efficacy among the groups analyzed. Therefore, we selected this group for further evaluation. Virus titers in lung tissues measured on 3 and 6 days p.i. are shown in Figure 3A. Virus titers in the lung decreased as a function of time in the 3D8 scFv-treated group but showed at high levels in the control group, compared with the 3D8 scFv pretreated group at 3 and 6 days p.i. (Figure 3A).

### 3.3. Reduced Influenza Virus Pathogenicity Due to the Preventive Effect of 3D8 scFv in the Lung

To confirm that the differences in clinical signs between the control and 3D8 scFv pretreated groups were due to a reduction in influenza virus pathogenicity, the expression levels of genes related to viral replication were evaluated by qRT-PCR using lung RNA samples obtained from the experiments described above. As shown in Figure 3B, expression of HA and NA mRNA was elevated in the control group, but significantly reduced in the 3D8 scFv pretreated group (Figure 3B). Consistent with the qRT-PCR results, immunohistochemistry showed that production of HA protein was inhibited in the 3D8 scFv pretreated group *versus* the control group (Figure 3C).

### 3.4. 3D8 scFv Reduced Histopathological Symptoms in Mouse Lung Tissue

We performed a histopathological examination by H&E staining to investigate the changes in cell shapes after virus infection. After H1N1 infection, the degree of infiltration of dense granulocytic and lymphocytic cells in the interstitium and around vessels and airways was less in the 3D8 scFv pretreated group than in the control group (Figure 3Df,l). Additionally, focally denuded lamina propria, attributed to epithelial necrosis and desquamation, was observed to a greater extent in the control group (Figure 3De,k).

### 3.5. 3D8 scFv Passes through the Nasal Mucosal Layer and Localizes in Epithelial Cells

**Figure 4:**
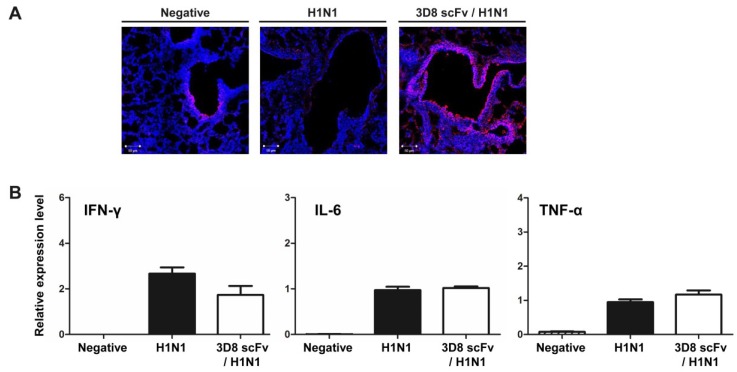
Penetration of 3D8 scFv into the epithelium of the nasal mucosa and analysis of cytokine and chemokine expression. (**A**) The presence of 3D8 scFv in the epithelium of the nasal mucosa of the lung was detected by immunohistochemistry. Lung tissues were stained with anti-3D8 scFv polyclonal Ab and visualized using a TRITC-conjugated anti-rabbit secondary Ab and fluorescence microscopy. Mice were treated with or without 3D8 scFv for 5 days and then challenged. After virus challenge, lung samples were extracted from each group on days 3 and 6 p.i.; (**B**) mRNA expression of the indicated cytokines and chemokines was measured by qRT-PCR with primers against IFN-β, IFN-γ, or GADPH.

To evaluate the correlations among the reduced histopathological signs, the decrease in viral gene expression and the presence of 3D8 scFv in respiratory epithelial cells, we assessed the localization of 3D8 scFv protein in epithelial cells by immunohistochemistry. 3D8 scFv protein was localized in medium-diameter bronchi and alveoli (Figure 4A). Specifically, strong immunohistochemical staining for 3D8 scFv was observed in the nasal layer, bronchus, and surrounding areas. These results indicated that 3D8 scFv passed through the nasal mucosal layer and penetrated the epithelial cells.

### 3.6. The Antiviral Effect in 3D8 ScFv-treated Mice is Due to Its Catalytic Activity against Nucleic Acids

To rule out an indirect antiviral effect of administration of 3D8 scFv administration through triggering an endogenous immune response, we examined the levels of host immune response genes by qRT-PCR. The levels of transcripts involved in the host pro-inflammatory response (TNF-α, IL-6) and known antiviral agents (IFN-γ) were increased in both the control group and the 3D8 scFv pre-treated group (Figure 4B). There was no statistically significant difference between the groups in the levels of expression of almost all of the transcripts analyzed. Thus, the antiviral effect of 3D8 scFv was not due to an indirect immune-related mechanism, but rather a direct result of its intrinsic RNase catalytic activity.

## 4. Discussion

Many types of influenza virus have been isolated and characterized in various countries around the world. In particular, recently, the H1N1 influenza virus recently emerged worldwide and caused global pandemic outbreaks in 2009 and 2013–2014. Many countries reported thousands of confirmed cases and several deaths caused by the H1N1 influenza virus [2,3,7,8,25]. Thus, there is a huge demand for direct preventive treatments for prevention against H1N1. In this report, we confirmed the preventive activity of 3D8 scFv against H1N1 influenza virus infection through its intrinsic RNase catalytic activity against the viral genome in both MDCK cells and a mouse model system. The expression level of viral genes (HA and NA) was significantly decreased in lung tissues of the 3D8 scFv-treated group (Figure 3B). The virus particle load was also decreased in the alveoli and bronchiole of lungs by immunohistochemistry (Figure 3C). These results suggest that 3D8 scFv can protect mice from H1N1 infection through inhibition of virus multiplication.

Consistent with the direct antiviral effects of 3D8 scFv reported in our previous studies [12,13,14], viral replication of PRV and HSV was directly inhibited by the intrinsic DNA and RNA hydrolyzing acitivity of 3D8 scFv in transgenic cell lines [12]. In a mouse model system, infection with PRV and MNV was prevented by endogenous expression of 3D8 scFv or by feeding with 3D8 scFv-expressing lactobacillus respectively [12,14]. Moreover, 3D8 scFv is able to directly inhibit PRV infection by intraperitoneal (i.p.) injection of purified protein (unpublished data). Therefore, the preventive activity against H1N1 infection observed in both MDCK cells and mice harboring 3D8 scFv expression in this study provide an example of the direct antiviral activity of 3D8 scFv against H1N1 influenza virus.

There have been numerous attempts to overcome the respiratory mucosal barrier for efficient delivery of systemically acting drugs. The possibility of using the respiratory tract for drug delivery has been demonstrated in mice for various vaccines against viruses such as influenza, herpes simplex virus, human immunodeficiency virus and human papillomavirus [26]. This approach could allow to access to the central nervous system (CNS) and local/systemic delivery [27]. However, these previous studies have not solved the limitation of protein drug transmission via the respiratory tract, especially for antibody therapies. There are only a few published studies on the therapeutic effects of monoclonal antibodies (mAb) delivered into the airways in animal models of pulmonary inflammation [28,29]. Intranasal administration of anti-IL-5 monoclonal antibody was shown to attenuate airway inflammation and hyperresponsiveness in a mouse model [29]. However, in these studies, the protective and neutralizing effect of the administered mAb was due to the stimulation of an indirect host immune response. In the current study, we tested the ability of 3D8 scFv to inhibit A/NWS/33 H1N1 infection via its intrinsic nuclease activity. We showed that 3D8 scFv penetrated into epithelial cells through the respiratory mucosal layer and finally spread broadly to the lung alveoli (Figure 4A). Furthermore, our immunohistochemistry data demonstrated that the localization of 3D8 scFv in the lung alveoli and epithelial cells could result in direct hydrolysis of the H1N1 genome and/or RNA transcripts through its intrinsic RNase activity. We observed a high level of transmission of 3D8 scFv into the lung tissues by immunohistochemistry but we did not determine the amounts of 3D8 scFv in epithelial cells quantitatively.

Many attempts have been made to increase the permeability and bioavailability of intranasally administered drug. In some cases, peptides or enhancers were fused to drugs. To overcome the difficulty of penetrating the nasal barrier, various other approaches have been utilized including modification of the permeability of nasal membranes by an absorption enhancer or the use of the mucoadhesive system such as a bioadhesive, liquid formulation and microsphere powder [30]. However, 3D8 scFv does not need any further enhancers or modifications to increase its permeability and bioavailability because it can penetrate into epithelial cells through respiratory mucosal layers. 3D8 scFv was previously characterized as a protein that can enter into cells by caveolae-mediated endocytosis [11]. Therefore, this ability of 3D8 scFv to penetrate epithelial cells and the respiratory mucosal layer provides potential as an effective intranasal drug candidate.

Overreaction of the host immune response (*i.e.*, a “cytokine storm”) induces a hyperinflammatory process and is involved in the pathogenicity of the influenza virus [31,32]. A tight correlation between the induction of genes involved in the inflammatory response and increased resistance against virus infection has been reported. Transcript levels of inducible nitric oxide synthase (iNOS) remain high in cells infected with several viruses, including HSV and influenza virus [33]. Similarly, levels of IL-6 and TNF-α correlated positively with lung inflammation and vascular dysfunction [32]. Thus, to determine whether the antiviral effect observed in our study was caused by direct RNase activity of 3D8 scFv or indirect activation of an endogenous antiviral response, we measured the expression levels of marker genes involved in the inflammation pathway. As shown in Figure 4B, levels of proinflammatory cytokines (IL-6, TNF-α) and antiviral proteins (IFN-γ) were significantly increased in the H1N1-infected group compared with the non-infected control group. However, the levels of transcripts of the H1N1-infected group were similar to those of the 3D8 scFv-treated group. These results indicated that the antiviral effects of 3D8 scFv against H1N1 infection were due to the direct intrinsic RNase activity of 3D8 scFv rather than a host immune response.

In conclusion, we demonstrated that the RNase activity of 3D8 scFv could be effectively employed *in vivo* as an antiviral agent against influenza virus by delivering the protein to the respiratory cavity via intranasal administration. The unique cell penetration properties of 3D8 scFv protein combined with its intrinsic RNase activity suggest that 3D8 scFv might have potential as a novel antiviral drug candidate.
